# Endoscopic findings and outcomes of gastric mucosal changes relating to potassium‐competitive acid blocker and proton pump inhibitor therapy

**DOI:** 10.1002/deo2.400

**Published:** 2024-06-24

**Authors:** Satoshi Shinozaki, Hiroyuki Osawa, Yoshimasa Miura, Hiroaki Nomoto, Hirotsugu Sakamoto, Yoshikazu Hayashi, Tomonori Yano, Edward J. Despott, Hironori Yamamoto

**Affiliations:** ^1^ Shinozaki Medical Clinic Tochigi Japan; ^2^ Department of Medicine Division of Gastroenterology Jichi Medical University Tochigi Japan; ^3^ Department of Medicine Division of Gastroenterology and Hepatology Nihon University School of Medicine Tokyo Japan; ^4^ Royal Free Unit for Endoscopy The Royal Free Hospital and UCL Institute for Liver and Digestive Health London UK

**Keywords:** endoscopy, gastroesophageal reflux disease, potassium‐competitive acid blocker, proton pump inhibitor, vonoprazan

## Abstract

Gastric mucosal changes associated with long‐term potassium‐competitive acid blocker and proton pump inhibitor (PPI) therapy may raise concern. In contrast to that for PPIs, the evidence concerning the safety of long‐term potassium‐competitive acid blocker use is scant. Vonoprazan (VPZ) is a representative potassium‐competitive acid blocker released in Japan in 2015. In order to shed some comparative light regarding the outcomes of gastric mucosal lesions associated with a long‐term acid blockade, we have reviewed six representative gastric mucosal lesions: fundic gland polyps, gastric hyperplastic polyps, multiple white and flat elevated lesions, cobblestone‐like gastric mucosal changes, gastric black spots, and stardust gastric mucosal changes. For these mucosal lesions, we have evaluated the association with the type of acid blockade, patient gender, *Helicobacter pylori* infection status, the degree of gastric atrophy, and serum gastrin levels. There is no concrete evidence to support a significant relationship between VPZ/PPI use and the development of neuroendocrine tumors. Current data also shows that the risk of gastric mucosal changes is similar for long‐term VPZ and PPI use. Serum hypergastrinemia is not correlated with the development of some gastric mucosal lesions. Therefore, serum gastrin level is unhelpful for risk estimation and for decision‐making relating to the cessation of these drugs in routine clinical practice. Given the confounding potential neoplastic risk relating to *H. pylori* infection, this should be eradicated before VPZ/PPI therapy is commenced. The evidence to date does not support the cessation of clinically appropriate VPZ/PPI therapy solely because of the presence of these associated gastric mucosal lesions.

## INTRODUCTION

The worldwide use of proton pump inhibitors (PPIs) is increasing and long‐term use of these drugs is also frequently required as maintenance therapy for gastroesophageal reflux disease (GERD). Long‐term use is also applied as a preventive strategy to reduce the risk of peptic ulceration and gastrointestinal bleeding in the context of non‐steroidal anti‐inflammatory drugs and low‐dose aspirin use.^1^, ^2^ Although *Helicobacter pylori* eradication decreases the risk of peptic ulceration, eradication success augments gastric acid secretion, and this may be associated with the occurrence of GERD. In Japan, the prevalence of *H. pylori* infection is decreasing, and this has been associated with a decrease in the prevalence of gastric atrophy.^3^ Active *H. pylori* infection is associated with diminished gastric acid secretion through associated dysfunction of parietal cells, and an increased presence of inflammatory cytokines within the gastric fundus. Therefore, *H. pylori* eradication increases gastric acid secretion through recovery of the H^+^/K^+^‐ATPase expression of parietal cells.^4^

PPI therapy suppresses gastric acid secretion through inhibition of H^+^/K^+^‐ATPase. Although three decades have passed since the introduction of PPI therapy into general clinical practice in the United States, there are still unmet needs for the effective management of GERD; this is mainly due to insufficient acid suppression. Acid blockade by PPIs is largely influenced by the CYP2C19 genotype status, and the pH 4 holding time of PPIs is only about 12 h in patients who are extensive metabolizers.^5^ Notably, even double‐dose PPI therapy fails to control nocturnal acid breakthrough symptoms.^6^ These patients also experience daytime heartburn because of an insufficient pH 4 holding time.^7^, ^8^ These failings have spurred research into seeking alternative, more robust acid blockade.

Vonoprazan (VPZ), a potassium‐competitive acid blocker (PCAB), was subsequently developed by Takeda Pharmaceutical Company Limited; it entered clinical practice in Japan in 2015. Unlike PPIs, VPZ works through direct and reversible H^+^‐K^+^ exchange in gastric parietal cells; its acid blockade is long‐lasting and not influenced by luminal acidity or the CYP2C19 genotype.^8^, ^9^, ^10^ Therefore, VPZ has a stronger and more stable acid suppressive effect as compared with PPIs. The intragastric pH 4 holding time is important in the treatment of GERD, *H. pylori* infection, and peptic ulcers. VPZ has been shown to have a significantly higher pH 4 holding time than esomeprazole or rabeprazole in *H. pylori*‐negative healthy volunteers.^7^, ^8^ VPZ has also been shown to be useful for long‐term control of symptomatic GERD.^11^, ^12^ It achieves superior outcomes over PPIs in patients with severe reflux esophagitis (Los Angeles grade C/D).^13^ A recent systematic review also reported the superior effect of VPZ on nocturnal acid breakthrough as compared with PPIs.^14^

To evaluate the long‐term safety of VPZ as compared with PPIs, the VISION (the Vonoprazan study In patients with eroSIve esophagitis to evaluate long‐term safety) study was conducted from 2016 to 2022. This open‐label randomized controlled prospective study included 135 patients treated with VPZ 10 or 20 mg once daily and 67 patients with lansoprazole 15 or 30 mg once daily for 5 years.^15^ The VISION study excluded patients with current infection of *H. pylori* or a history of *H. pylori* eradication, and therefore we assume that patients with gastric atrophy were mostly excluded. The VISION study performed a histopathological evaluation of biopsies from included patients on a yearly basis.^15^

Given the increasing concerns associated with long‐term acid suppression therapy, it is important to scientifically evaluate and understand the prevalence and features of any associated gastric mucosal changes. In contrast to that for PPIs, the evidence concerning the safety of long‐term PCAB use is scant. Although PCABs also include the drug tegoprazan,^16^ any long‐term follow‐up data only relate to the use of VPZ. Therefore, in this review, we have focused on the long‐term safety and gastric mucosal changes associated with VPZ and PPI use (Table 1).

**TABLE 1:** Six representative gastric lesions associated with vonoprazan /proton pump inhibitors.

	Prevalence			Gender	*H. pylori*	Gastric atrophy	Hypergastrinemia
	VPZ user	PPI user	Comparison				
Fundic gland polyps	43%–72%	14%–85%	VPZ < PPI	M < F	No infection	None to mild	Not related
Gastric hyperplastic polyps	8%–23%	9%–12%	VPZ > PPI	M = F	Current infection	Moderate to severe	Related?
Multiple white and flat elevated lesions	8%–30%	13%–49%	VPZ < PPI	M < F	History of eradication	?	Not related
Cobblestone‐like gastric mucosal changes	9%–22%	11%–35%	VPZ = PPI	?	?	None to mild	Not related
Gastric black spots	8%	13%–16%	VPZ < PPI	?	History of eradication	?	Not related
Stardust gastric mucosal changes	5%–68%	11%–12%	VPZ > PPI	M < F	?	None to mild	Related?

Abbreviations: F, female; *H. pylori*, *Helicobacter pylori*; M, male; PPI, proton pump inhibitor; VPZ, vonoprazan.

### Histopathology of the stomach during VPZ/PPI therapy

Long‐term PPI use promotes cystic dilation of fundic glands and foveolar epithelial hyperplasia.^17^ Enterochromaffin‐like (ECL) cell proliferation caused by long‐term PPI use is also well demonstrated by a systematic review.^18^ This same systematic review showed no evidence of the development of dysplastic or neoplastic changes in association with PPI use.^18^ Parietal cell protrusion is recognized as prominent intraluminal protrusions, and is associated with a narrow and serrated glandular lumen (sparse secretory canaliculi).^19^ These changes are proportionate to the duration of PPI therapy. In the VISION study, any ECL cell hyperplasia was similar for the VPZ and PPI groups at 5 years.^20^

### Fundic gland polyps

**FIGURE 1:**
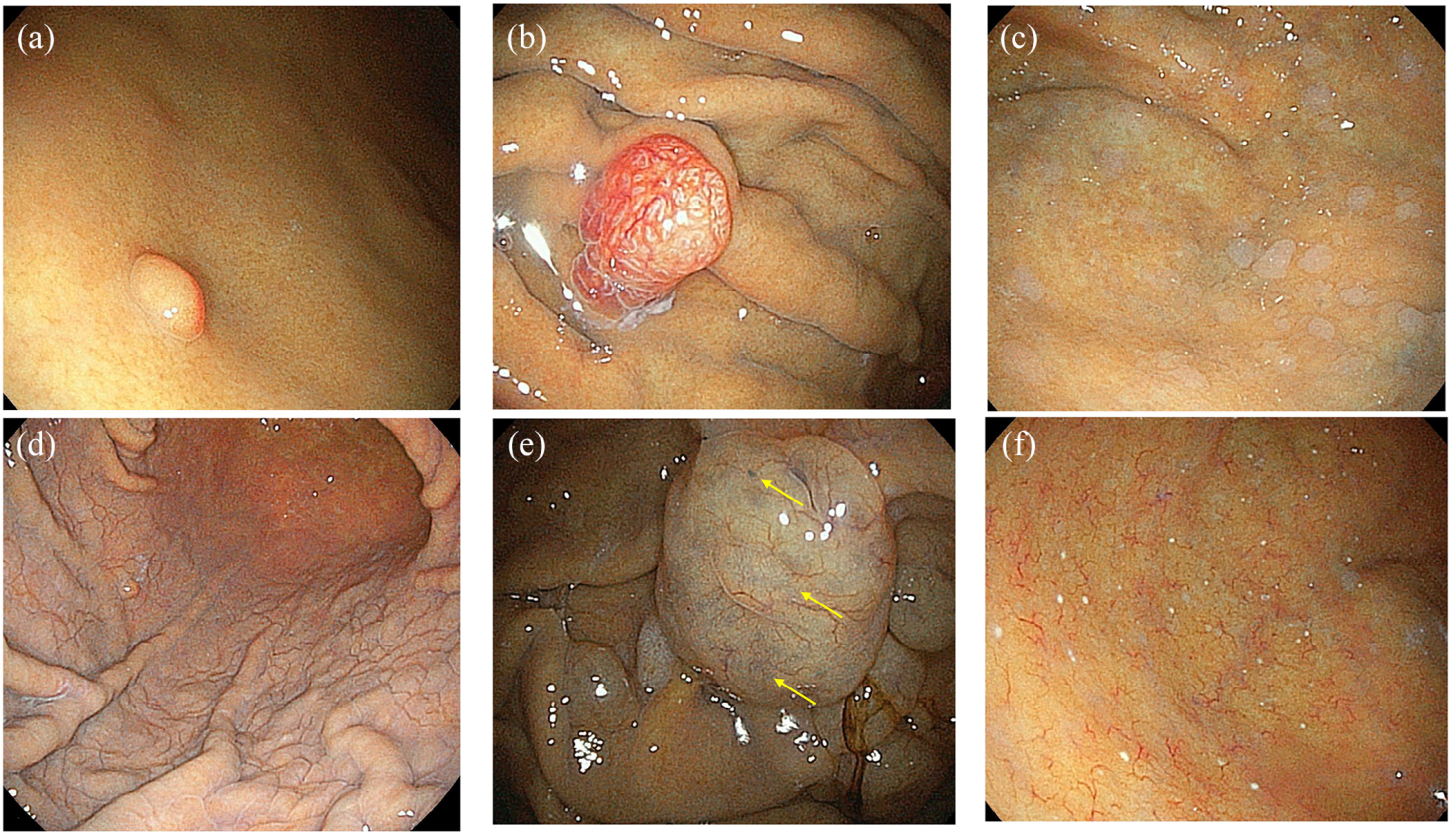
Six representative gastric mucosal changes associated with vonoprazan/proton pump inhibitors: (a) fundic gland polyp, (b) gastric hyperplastic polyp, (c) multiple white and flat elevated lesions, (d) cobblestone‐like gastric mucosal changes, (e) gastric black spots (arrows), and (f) stardust gastric mucosal changes.

Fundic gland polyps account for 77% of all gastric polyps.^21^ Fundic gland polyps usually develop in multiple sites on the gastric fundus and corpus and have a sessile morphology with a smooth translucent surface (Figure 1a). The association of long‐term PPI therapy and the development of fundic gland polyps is well described and these are recognized as the most frequent gastric mucosal lesions associated with PPI use. In a prospective study including 1,780 patients from Argentina, 4.3% had fundic gland polyps; 64% of patients with fundic gland polyps were on PPI therapy.^22^ A meta‐analysis including 12 studies reported that the duration of PPI therapy is proportionate to the prevalence of fundic gland polyps.^23^ Jalving et al. also reported that PPI administration for >5 years increases the prevalence of fundic gland polyps; cystic changes in these polyps were more prevalent in PPI users.^24^

The histological hallmark of fundic gland polyps associated with acid blocker use is cystic gland dilation due to parietal cell protrusion and foveolar cell proliferation.^25^ The underlying etiological pathway of this association with acid blockade is as yet unclear, but protrusion and hyperplasia of parietal cells are always present and are considered to be the precursor of these changes.^19^ Parietal cell protrusion (swelling and bulging of parietal cells) within the neck of fundic glands causes narrowing of secretory canaliculi, which may in turn cause retention of hydrochloric acid, resulting in the formation of fundic gland cysts.^26^ The elevated intraglandular pressure caused by increased resistance to outflow enlarges fundic gland cysts and develops within the first month of PPI therapy.^26^

A Japanese prospective study reported that the developmental rate of fundic gland polyps after starting PPI was 6.3% and 13.6% at 1 and 2 years, respectively; however, this study included a large population of *H. pylori*‐positive subjects (41%).^27^ Ongoing *H. pylori* infection reduces the prevalence of fundic gland polyps. We evaluated the prevalence of fundic gland polyps in patients treated with VPZ or PPI,^28^, ^29^ and found these to be significantly more prevalent than in our control group (*p* < 0.001). Furthermore, the prevalence was significantly higher in the PPI group than in the VPZ group (*p* = 0.008). The prevalence of fundic gland polyps >10 mm in size was also significantly higher in the PPI group as compared with the VPZ and the control groups.^29^ The results of the VISION study supported our aforementioned results and showed that the prevalence of fundic gland polyps in the PPI group was higher than in the VPZ group.^20^ As expected, considering its more potent acid suppression, serum gastrin levels are higher with VPZ as compared with PPI therapy. Although a correlation between fundic gland polyp formation and PPI use is well documented, it does not appear to be influenced by the associated hypergastrinemia. Two Japanese studies reported that there was no significant relationship between serum gastrin level and fundic gland polyp formation.^27^, ^30^ This is echoed by the findings of another European study.^31^ Therefore, we hypothesize that fundic gland polyp formation associated with VPZ/PPI is influenced by a gastrin‐independent pathway.

PPI therapy increases parietal cell proliferation via elevated expression of aquaporin‐4.^32^ In a mouse model, lansoprazole administration has been associated with histological findings similar to those of the fundic gland polyp, including multiple cystic dilatations and an increase in the number of water channel, aquaporin‐4 and potassium channel, KCNQ1‐positive parietal cells.^32^ The function of aquaporins is to work as channels to transport water across cell membranes, in response to osmotic gradients.^33^ A hemorrhagic fundic gland polyp associated with long‐term PPI administration was found to have aquaporin‐4 and KCNQ1‐positive parietal cells.^34^ The aquaporin‐4 positive parietal cells increased by PPI therapy enhance water transport into parietal cells, resulting in an enhanced influx of water from the interstitial space into the oxyntic glands.^35^ Distribution of aquaporin‐4 and KCNQ1 during acid suppression therapy may explain the difference in the prevalence of fundic gland polyps between VPZ and PPI groups.

Although excessive enlargement of fundic gland polyps may cause bleeding, an increased number of fundic gland polyps is not considered to be of clinical concern, since their malignant potential is reported to be negligible.^24^ It is not mandatory to cease acid blocker therapy due to the formation and/or increase of fundic gland polyps unless they cause bleeding or show signs of dysplasia. The subject merits further study.

### Gastric hyperplastic polyps

Gastric hyperplastic polyps are usually reddish and sessile and tend to form within the corpus and antrum (Figure 1b); they may be associated with atrophic gastritis caused by persistent *H. pylori* infection.^36^ Their histopathology is characterized by excessive proliferation of the foveolar epithelium caused by chronic inflammation of the gastric mucosa. Successful eradication of *H. pylori* results in complete regression of gastric hyperplastic polyps in approximately 44%–80% of patients.^37^, ^38^, ^39^ Growth of these lesions may result in spontaneous bleeding and malignant transformation.

Long‐term PPI administration is also associated with the development of gastric hyperplastic polyps. A Japanese prospective study and the VISION study found a similar prevalence of gastric hyperplastic polyps at 2 years of administration of PPIs (8.9% and 11.3%, respectively).^27^ At 5 years follow‐up, the VISION study showed that the prevalence of these lesions in the VPZ group was higher than that of the PPI group (23% vs. 11%).^20^ We previously reported a prevalence of biopsy‐proven gastric hyperplastic polyps in our VPZ and PPI groups (8% and 12%, respectively), the prevalence in both these groups was significantly higher than that of the control group (1%).^28^ Multivariate analysis showed that the presence of gastric hyperplastic polyp was significantly associated with both VPZ and PPI use.^28^

A Japanese study reported a positive correlation between the development of gastric hyperplastic polyps and serum gastrin levels.^27^ However, these findings were confounded by the inclusion of a high percentage of *H. pylori*‐positive subjects (41%).^27^ Although immunohistochemical staining of PPI‐associated gastric hyperplastic polyps shows expression of gastrin receptors in the focal foveolar epithelium,^40^ the exact causal relationship between hypergastrinemia and the development of gastric hyperplastic polyps remains unclear.

Malignant transformation of gastric hyperplastic polyps remains a concern during VPZ/PPI therapy. Ahn et al. showed that dysplasia/carcinoma was present in 3.7% of excised gastric hyperplastic polyps >1cm in size; loss of p16 expression and high Ki‐67 expression were observed in dysplastic areas of these lesions.^41^ However, this study was again confounded by the inclusion of a percentage of patients with ongoing *H. pylori* infection (61%); furthermore, the proportion of patients on PPI therapy was not reported. Therefore, carcinogenesis may be influenced by *H. pylori*‐related inflammation rather than acid blockade. Hizawa et al. also reported that 3.4% of resected gastric hyperplastic polyps had neoplastic components.^42^ Immunohistochemical analyses of six gastric hyperplastic polyps (1–4 cm in size) with malignant transformation indicated multistep carcinogenesis such as hyperplasia‐adenoma (dysplasia)‐adenocarcinoma sequence.^43^ Given the confounding factors of these studies, to date, there is no rigorous evidence to support malignant change in gastric hyperplastic polyps associated with VPZ/PPI use.

In order to assess for neoplastic transformation in gastric hyperplastic polyps, complete excision is advised, since biopsies alone are insufficient to exclude malignant change, as this may be limited to a tiny area of the lesion. PPI‐induced gastric hyperplastic polyps usually regress after cessation of PPI therapy.^40^, ^44^, ^45^ If gastric hyperplastic polyps <1 cm developed during VPZ/PPI therapy, cessation of VPZ/PPI is not mandatory. However, gastric hyperplastic polyps >1 cm should be endoscopically resected when they do not regress even with cessation of VPZ/PPI.

### Multiple white and flat elevated lesions

Multiple white and flat elevated mucosal lesions spreading from the fundus to the corpus were first reported by Kawaguchi et al. in 2007.^46^ Endoscopic findings are well‐circumscribed, round, slightly elevated, multiple lesions with smooth surfaces accompanied by white tubular structures (Figure 1c). Linked color imaging, one of the image‐enhanced endoscopic optical modalities developed by Fujifilm Medical facilitates visualization of these lesions. Their histopathology is characterized by foveolar epithelial hyperplasia accompanied by parietal cell protrusions and fundic gland dilatations.^47^, ^48^ In 2018, Hasegawa et al. reported that multiple white and flat elevated lesions were associated with PPI use.^48^ However, multivariate analysis in another Japanese study including 1995 subjects showed a significant association with female gender, advanced age, and moderate to severe gastric atrophy, but not with acid blocker use.^49^ Majima et al. reported the prevalence of multiple white and flat elevated lesions as 10.4% and multivariate analysis identified advanced age, female gender, and PPI use as positive significant predictors.^50^ In another, recent Japanese study published in 2023, the multivariate analysis identified PPI use as the only significant factor for these lesions.^47^

The VISION study showed slightly higher prevalence in the PPI group than in the VPZ group at 5 years after starting acid suppression therapy (14.9% vs. 8.9%).^20^ This study did not include patients with a history of *H. pylori* eradication therapy, and therefore we may assume that patients with severe gastric atrophy were mostly excluded. We previously also reported on the prevalence of multiple white and flat elevated lesions. Our PPI group had a significantly higher prevalence as compared with the VPZ group (40% vs. 30%, *p* = 0.027); multivariate analysis identified advanced age, female gender, PPI use, and VPZ use as positive predictors.^28^

Although there is a difference in prevalence rates of multiple white and flat elevated lesions among studies, it may be explained by the difference in the use of image‐enhanced endoscopies such as linked color imaging, blue laser imaging (BLI; Fujifilm Medical) and narrow‐band imaging (NBI, Olympus Medical) and/or the duration of VPZ/PPI use. Two Japanese studies including the VISION study and our retrospective study showed a similar tendency for PPI users to have more multiple white and flat elevated lesions than VPZ users.^20^, ^28^ VPZ users had significantly stronger acid suppression and higher serum gastrin levels as compared with PPI users.^7^ Multiple white and flat elevated lesions are frequently observed in patients with successful *H. pylori* eradication and possibly decreased serum gastrin levels.^49^ A Japanese study did not show a significant difference between serum gastrin levels and these lesions.^30^ We may therefore surmise that serum gastrin levels may not influence the development of these lesions. The malignant potential of multiple white and flat elevated lesions may be negligible.^50^ Even if multiple white and flat elevated lesions develop during VPZ/PPI therapy, cessation of clinically appropriate VPZ/PPI use is not necessary.

### Cobblestone‐like gastric mucosal changes

Cobblestone‐like mucosal changes are characterized by innumerable, slightly elevated lesions that are <5 mm in size; it is also known as ‘cracked gastric mucosa’ (Figure 1d).^17^, ^40^ The histopathological findings are those of oxyntic gland dilation, cytoplasmic vacuolation, and parietal cell protrusion.^40^, ^51^, ^52^ The visualized mucosal elevation may be caused by this oxyntic gland dilation.

A Japanese study reported the prevalence of cobblestone‐like gastric mucosal changes to be 3.4%, with the multivariate analysis identifying diabetes mellitus and PPI use as significant associated factors; treatment duration of PPI was not correlated with prevalence.^53^ Another Japanese study confirmed these findings, and showed a significantly higher prevalence of cobblestone‐like mucosal changes in PPI users as compared with patients who were not on PPI therapy (24.4% vs. 3.7%, *p* < 0.01); treatment duration was also not found to be an independent predictor.^40^ Interestingly, none of the patients with cobblestone‐like mucosal changes had active *H. pylori* infection, and most of these did not have any gastric atrophy.^40^ In the cohort of 171 patients who had been treated with PPIs for > six months, 35% were found to have cobblestone‐like mucosal changes.^52^ Cobblestone‐like mucosal change was also found to be more frequent in dialysis patients with associated hypergastrinemia^52^; however, elevated gastrin levels in dialysis patients is mainly caused by ‘big gastrin’ (G‐34), which has a much weaker function than ‘little gastrin’ (G‐17).^54^ Patients with cobblestone‐like gastric mucosal changes tend to lack gastric mucosal atrophy. While the serum gastrin level is proportionate to the degree of gastric atrophy,^52^, ^55^ the precise mechanism of the development of a cobblestone‐like mucosal change is unclear. Although the association of cobblestone‐like mucosal changes with hypergastrinemia is worthy of attention, two studies did not show a direct correlation with its presence.^30^, ^40^ Therefore, as yet unknown, alternative causative pathway, is more likely to be at play.

The presence of cobblestone‐like mucosal changes in patients treated with VPZ was first reported by Miyamoto et al.^51^ At 5‐year follow‐up, the VISION study also reported its presence in 22.1% and 15.1% in the VPZ and PPI groups, respectively.^20^ In our previous study,^28^ the prevalence in the VPZ and PPI groups was 9% and 12%, respectively, and these rates were significantly higher than that of the control group (0.6%).^28^ Multivariate analysis identified male gender, VPZ, and PPI use as significant associated factors.^28^ Further studies are necessary to understand the development of cobblestone‐like mucosal changes, its association with the acid blockade, and any clinical significance.

### Gastric black spots

This comparatively rare lesion was first reported in 2016 by Hatano et al.^56^ It is more likely to occur in patients with long‐term PPI treatment who have been on the drug for more than 1 year. The prevalence was reported as 0.24% in routine esophagogastroduodenoscopy, and all black spots were located on the fundic gland region (Figure 1e). Black spots are present on fundic gland polyps or the flat mucosa, and they are histologically characterized by parietal cell protrusions, fundic gland cysts, and brownish pigmentation in the fundic gland cysts resulting from the accumulation of mucus. Therefore, gastric black spots are also considered to be a gastric mucosal change associated with PPI use.

In contrast to the aforementioned study,^56^ Adachi et al. reported a higher prevalence of gastric black spots, of the order of 9.8% (156/1600).^57^ This study identified advanced age and a history of *H. pylori* eradication as significant associated factors, but not PPI use. Of these patients with black spots, 92% had a history of *H. pylori* eradication.^57^ Another Japanese study including 1214 patients reported the prevalence of black spots to be 6.2%; multivariate analysis identified low body mass index, a history of *H. pylori* eradication and PPI use as significant factors.^53^ Therefore, the presence of black spots may be considered a hallmark of successful *H. pylori* eradication. The VISION study found the prevalence of black spots in VPZ and PPI users at 5‐year follow‐up to be 7.7% and 13.2%, respectively.^20^

The precise mechanism for the development and prevalence of black spots remains elusive, and its association with VPZ/PPI use is also unclear. Although association with hypergastrinemia was reported,^30^ the VISION study^20^ did not support this association, since black spots were found more frequently in PPI users than in VPZ users. It is therefore likely that hypergastrinemia may have little influence on the development of gastric black spots. Further larger studies are warranted to clarify the association of VPZ/PPI therapy and any clinical relevance of gastric black spots.

### Stardust gastric mucosal changes

The detailed endoscopic and pathological images of stardust gastric mucosal lesions were first reported as “white globe appearance” that contains intraglandular necrotic debris.^59^, ^60^ After the release of VPZ, the presence of multiple small white protrusions, located within the fundus and corpus as observed at esophagogastroduodenoscopy was reported in 2020 by Yoshizaki et al.^58^ Due to its appearance, this was termed ‘stardust gastric mucosa’ (Figure 1f). The prevalence rates in VPZ and non‐VPZ users were 4.9% and 0.2%, respectively. The histopathological features are of a mucus pool within a dilated duct surrounded by flattened glandular epithelium, accompanied by parietal cell protrusion/vacuolation.^30^, ^58^ In multivariate analysis, the risk factors were found to be female gender and long‐term VPZ use. Both the prevalence rate and the number of lesions were proportionate to the duration of VPZ therapy.^58^

In 2021, Nishiyama et al. termed the stardust gastric mucosal changes as “white spots”.^30^, ^61^ There was a higher prevalence in the group of patients on acid blockade therapy than that of the control group (27.5% vs. 1.3%, respectively, *p* < 0.001). The VPZ group showed a significantly higher prevalence than the PPI group (68% vs. 12%, *p* < 0.001). The serum gastrin level was significantly higher in patients with stardust gastric mucosal changes than in those without this finding. In VPZ users, patients with stardust gastric mucosal changes had significantly higher serum gastrin levels than those without (*p* < 0.05). The multivariate analysis identified high serum gastrin level and VPZ use as associated factors.

In 2022, we reported on the prevalence of stardust gastric mucosal changes.^28^ The prevalence was 48%, 11%, and 0.5% in the VPZ, PPI, and control groups, respectively. The prevalence in the VPZ and PPI groups was significantly higher than that of the control group (*p* < 0.001); the VPZ group had a significantly higher prevalence than the PPI group. In multivariate analysis, both VPZ and PPI use were identified as positive significant factors associated with stardust gastric mucosal change. Female gender was also a significant associated factor. Therefore, stardust gastric mucosal change is not a specific lesion associated solely with VPZ therapy.

In the latter two studies, the prevalence rates were similar,^28^, ^61^ and much higher than that of the first report.^58^ We hypothesize that the reported difference in prevalence may be explained both by the duration of acid suppression therapy and the endoscopists’ subsequent heightened awareness of this finding. Although a correlation between hypergastrinemia and stardust gastric mucosal change was reported, a causal relationship is not evident. Although concerns were raised regarding the possible associated development of neuroendocrine neoplasia,^62^ these are allayed by the fact that stardust gastric mucosal change is also observed in patients treated with PPIs, drugs that have been safely in us for over 30 years. Although there is no evident association with neoplastic change, further large studies are necessary to elucidate the mechanism of development of stardust gastric mucosal change and its long‐term natural history.

### Gastric atrophy

The presence of gastric atrophy has been definitively associated with an increased risk of gastric cancer.^63^ Although a possible association between PPI use and gastric atrophy has been discussed, the pooled analysis including 1070 participants from four randomized‐controlled trials showed no statistically significant differences in risk between PPI users and non‐PPI users (odds ratio 1.50, 95% confidence interval 0.59–3.80, *p* = 0.39).^18^ Long‐term PPI use is not associated with the development of intestinal metaplasia.^18^ A recent randomized controlled trial with 3 years of follow‐up did not demonstrate a significant association between PPI use and gastric atrophy.^64^ Perhaps surprisingly, the 3‐year interim analysis of the VISION study showed improvement in antral gastric atrophy in both the VPZ and PPI groups.^65^ Therefore, we believe that the progression of gastric atrophy is mainly influenced by *H. pylori* infection status, rather than acid blockade.

### Hypergastrinemia

Gastrin, secreted by the G cells within the gastric antrum is an important hormone for modulation of gastric acid secretion through the regulation of ECL cell function. The hypoacidity caused by acid suppression therapy stimulates gastrin secretion through negative feedback, and this results in ECL cell hyperplasia. In animal models, ECL cell density has been found to be proportionate to the serum gastrin level, and efficient inhibition of gastric acid secretion is accomplished through its maximal trophic effect.^66^ In humans, hypergastrinemia also induces a trophic effect on the oxyntic mucosa and ECL cells.^67^ However, concern about long‐term acid suppression therapy with VPZ/PPI, has led to a focus on serum gastrin level measurement, regardless of the unknown clinical significance of its elevation in the context of long‐term acid blockade therapy.

A Japanese study including 382 patients reported that PPI use, corpus atrophy, and female gender were identified as positive significant factors for hypergastrinemia in multivariate analysis.^68^ In the VISION study, the serum gastrin level of the PPI and VPZ groups during the 5‐year treatment period ranged approximately from 300–400 to 700–900 pg/mL, respectively, and the values were significantly higher than the baseline (approx. 130–150).^20^ However, the proportion of patients with ECL‐cell hyperplasia was similar between VPZ and PPI groups at 5 years of follow‐up (VPZ 4.9% vs. PPI 7.7%, respectively).^20^

In our retrospective study excluding patients with current *H. pylori* infection published in 2022, we reported the 4‐year changes in serum gastrin level in patients continuously treated with VPZ.^55^ Overall, the serum gastrin level for 4 years was in the range of 700–1200 pg/mL; this plateaued at 1.5 years and was maintained for 2.5 years without further elevation.^55^ This long‐term change was similar to the findings of a previous study of patients on PPI therapy, where serum gastrin level plateaued at about 1–2 years and was maintained for 3–4 years.^69^ In our VPZ study, multivariate analysis revealed that severe gastric atrophy was associated with hypergastrinemia. The gastrin level over 4 years in patients with severe gastric atrophy and those with no atrophy was in the range of 900–1500 and 500–1000 pg/mL, respectively.^55^ The latter value was similar to the serum gastrin value observed in the VISION study which mostly included patients without gastric atrophy.^20^ In a multicenter prospective study, the VPZ group showed significantly higher serum gastrin levels than the PPI group, in patients with no or mild gastric atrophy.^70^ However, no significant difference between both groups was observed in patients with severe gastric atrophy.

The clinical significance of long‐term hypergastrinemia caused by acid blockers is still unclear, and serum gastrin level can be influenced by multiple factors including gastric atrophy, chronic kidney disease, glucocorticoid therapy, as well as VPZ/PPI use.^71^ In a recent study evaluating the human gastric mucosa, no relationship was found between neuroendocrine markers and hypergastrinemia secondary to long‐term PPI therapy.^72^ An increase in serum gastrin level alone is therefore not considered to be a valid reason for the cessation of clinically appropriate long‐term VPZ/PPI therapy. Periodical measurement of serum gastrin levels is discouraged since it is neither necessary nor helpful for decision‐making in routine clinical practice.

### Gastric neuroendocrine neoplasm

The potential development of neuroendocrine tumors during acid suppression therapy has always been of concern. The development of such tumors requires multistep carcinogenesis commencing at ECL cell hyperplasia. A systematic review showed that ECL cell hyperplasia is significantly associated with PPI use.^18^ However, there is no rigorous evidence to suggest that VPZ/PPI administration increases the risk of gastric neuroendocrine tumor development; this is supported by the Japanese Guidelines.^73^ Since VPZ users have significantly higher serum gastrin levels as compared with PPI users,^20^ the potential development of neuroendocrine neoplasm via ECL cell hyperplasia during VPZ remained of concern. The VISION study evaluated gastric biopsies at 5 years of follow‐up since the start of VPZ/PPI therapy and reassuringly showed a similar rate of ECL cell hyperplasia (1‐6%) without the development of neuroendocrine tumors.^20^ Although there are anecdotal reports indicating neuroendocrine tumor development during VPZ therapy,^74^ there are no studies demonstrating a significant relationship between VPZ use and neuroendocrine tumor formation.

*H. pylori* eradication success can decrease serum gastrin levels and is therefore strongly recommended before commencing VPZ/PPI therapy. If the development of a neuroendocrine tumor is confirmed, VPZ/PPI treatment should be discontinued. VPZ/PPI administration should be avoided in patients with type A gastritis because of its associated strong hypergastrinemia and high risk of neuroendocrine tumor formation. A Japanese multicenter retrospective study reported a favorable prognosis in patients with type 1 gastric carcinoids during long‐term follow‐up (disease‐specific survival: 100%).^75^ Even if type 1 gastric neuroendocrine tumors develop, their presence will not influence overall survival as long as these are appropriately managed. Nevertheless, we cannot eliminate the relationship between acid blockade and the development of gastric neuroendocrine tumors. Since carcinogenesis (as is also seen in the case of *H. pylori*) may take several decades to develop, long‐term observation for more than 10 years is necessary to fully understand any potential associated risk for the development of gastric malignancy. The present evidence is therefore still not sufficient to rule out any potential risk of malignancy associated with long‐term administration of VPZ/PPI.

## CONCLUSION

The risk of gastric mucosal change is similar for long‐term VPZ and PPI use. Hypergastrinemia is not correlated with the development of fundic gland polyps, gastric black spots, and multiple white and flat elevated lesions. Therefore, serum gastrin level is unhelpful in routine clinical practice and cannot serve as a guide for the cessation of VPZ/PPI therapy. To avoid a confounding factor that is intrinsically associated with malignant risk, patients with *H. pylori* infection should be excluded from future studies on this subject. It is also essential to ensure that *H. pylori* eradication is successfully achieved before the commencement of VPZ/PPI therapy. The development of the aforementioned gastric mucosal lesions alone during VPZ/PPI therapy is not considered a justified reason for the cessation of clinically appropriate VPZ/PPI therapy. Although such mucosal changes during long‐term use of VPZ/PPI should be observed, the benefits of clinically appropriate acid blockade presently outweigh any currently understood risks. However, the matter merits further longitudinal, follow‐up studies to confirm the long‐term safety of prolonged therapy with these agents.

## CONFLICT OF INTEREST STATEMENT

Satoshi Shinozaki, Hiroyuki Osawa, and Yoshimasa Miura have received honoraria from Takeda and Otsuka Pharmaceuticals. Hirotsugu Sakamoto, Tomonori Yano, and Hironori Yamamoto have received honoraria from Takeda Pharmaceutical. All other authors declare no conflict of interest.

## ETHICS STATEMENT

Not applicable.
